# Vitamin D treatment during pregnancy prevents autism-related phenotypes in a mouse model of maternal immune activation

**DOI:** 10.1186/s13229-017-0125-0

**Published:** 2017-03-07

**Authors:** Stephanie Vuillermot, Wei Luan, Urs Meyer, Darryl Eyles

**Affiliations:** 1 Swiss Federal Institute of Technology (ETH) Zurich, 8603 Schwerzenbach, Switzerland; 2 Queensland Brain Institute, The University of Queensland, Brisbane, 4072 Queensland Australia; 3 Institute of Pharmacology and Toxicology, University of Zurich-Vetsuisse, Zurich, Switzerland; 4 Queensland Centre for Mental Health Research, Brisbane, Queensland 4076 Australia

**Keywords:** Maternal immune activation, Vitamin D, Neurodevelopmental disorders, Schizophrenia, Autism, Cytokines, Dopamine

## Abstract

**Background:**

Prenatal exposure to infection is a recognized environmental risk factor for neuropsychiatric disorders of developmental origins such as autism or schizophrenia. Experimental work in animals indicates that this link is mediated by maternal immune activation (MIA) involving interactions between cytokine-associated inflammatory events, oxidative stress, and other pathophysiological processes such as hypoferremia and zinc deficiency. Maternal administration of the viral mimic polyriboinosinic-polyribocytidylic acid (poly(I:C)) in mice produces several behavioral phenotypes in adult offspring of relevance to autism spectrum disorder (ASD) and other neurodevelopmental disorders.

**Methods:**

Here, we investigated whether some of these phenotypes might also present in juveniles. In addition, given the known immunomodulatory and neuroprotective effects of vitamin D, we also investigated whether the co-administration of vitamin D could block MIA-induced ASD-related behaviors. We co-administered the hormonally active form of vitamin D, 1α,25 dihydroxy vitamin D3 (1,25OHD), simultaneously with poly(I:C) and examined (i) social interaction, stereotyped behavior, emotional learning and memory, and innate anxiety-like behavior in juveniles and (ii) the levels of the pro-inflammatory cytokines IL-1β, IL-6 and TNF-α in maternal plasma and fetal brains.

**Results:**

We show that like adult offspring that were exposed to MIA, juveniles display similar deficits in social approach behavior. Juvenile MIA offspring also show abnormal stereotyped digging and impaired acquisition and expression of tone-cued fear conditioning. Importantly, our study reveals that prenatal administration of 1,25OHD abolishes all these behavioral deficits in poly(I:C)-treated juveniles. However, prenatal administration of vitamin D had no effect on pro-inflammatory cytokine levels in dams or in fetal brains suggesting the anti-inflammatory actions of vitamin D are not the critical mechanism for its preventive actions in this ASD animal model.

**Conclusions:**

This work raises the possibility that early dietary supplementation with vitamin D may open new avenues for a successful attenuation or even prevention of neurodevelopmental disorders following maternal inflammation during pregnancy.

**Electronic supplementary material:**

The online version of this article (doi:10.1186/s13229-017-0125-0) contains supplementary material, which is available to authorized users.

## Background

Prenatal exposure to infection is a recognized environmental risk factor for neuropsychiatric disorders of developmental origins. This epidemiological association has been most widely studied in the context of schizophrenia [1]. Since the first report in 1971 [2], however, evidence has accumulated to suggest that prenatal infection also is a risk factor for autism spectrum disorder (ASD) [3, 4]. For example, studies of the Danish health registry show that from more than one million children born between 1980 and 2005 there was an almost threefold increase in the rate of ASD diagnosis in children born to mothers who were hospitalized for viral infection during pregnancy [5]. A similar (albeit somewhat less strong) association has been found in a large Swedish nationwide register-based birth cohort born 1984–2007 with follow-up through 2011 [6]. Experimental work in animals indicates that this link is mediated by maternal immune activation (MIA) involving an interplay between cytokine-associated inflammatory events [7–9], oxidative stress [10], and other pathophysiological processes such as hypoferremia [11] and zinc deficiency [12]. The centrality of inflammatory cytokine production to MIA phenotypes is supported by the findings that blocking the actions of pro-inflammatory cytokines such as IL-1β, IL-6, or IL-17a in the pregnant maternal host is capable of preventing the long-term brain and behavioral consequences of prenatal immune activation [7, 9, 13, 14]. Similarly, over-expression of the anti-inflammatory cytokine IL-10 prevents the emergence of multiple behavioral and pharmacological abnormalities typically seen after prenatal immune challenge [8].

Currently, the most widely used animal model for MIA is gestational exposure to the viral mimic, polyriboinosinic-polyribocytidylic acid (poly(I:C)). Poly(I:C) is a synthetic analog of double-stranded RNA that induces a cytokine-associated viral-like acute phase response in maternal and fetal compartments, including the fetal brain [15]. Initially developed in the context of preclinical schizophrenia research [16–18], more recent research shows that this model also reproduces an array of adult behavioral phenotypes with relevance to ASD, including impairments in social interaction and species-specific communication, stereotyped behaviors, and altered emotional learning and behavior [9, 19–21]. Hence, poly(I:C)-based MIA models provide experimental support for epidemiological findings linking prenatal immune challenges with increased risk of ASD [2, 5, 6]. One limitation of current MIA models, however, is that they mostly focused on ASD-related phenotypes once the offspring reached early adulthood, whereas overt ASD symptoms typically emerge in children.

Another epidemiologically valid developmental risk factor for later psychiatric disease is developmental vitamin D (DVD) deficiency [22]. Besides its role in schizophrenia etiology, there is a growing body of evidence linking vitamin D deficiency with autism [23]. Birth cohort studies have provided evidence that maternal vitamin D deficiency is associated with a range of later autism-related outcomes including impaired language development [24] and cognitive development in offspring [25]. Most recently, we have shown that low levels of vitamin D at birth are associated with increased incidence of autism in children [26]. Low vitamin D would also appear to be prevalent in children diagnosed with autism [27].

In addition to its classical role in calcium and bone homeostasis, vitamin D has major immunomodulatory roles associated with inflammation [28]. For instance, vitamin D interferes with pro-inflammatory transcription factors and signaling pathways, regulates the expression of pro-inflammatory enzymes, and modulates cytokine gene expression, protein production, and signaling. More specifically, vitamin D inhibits the production of pro-inflammatory cytokines such as IL-6 or TNF-alpha in monocytes via the inhibition of p38 MAP kinase [29], and it downregulates the expression of IL-6 mRNA [30]. Vitamin D also reduces the release of IL-1β in stimulated peripheral blood mononuclear cells (PBMC) [31]. Developmental vitamin D deficiency has further been shown to induce persistent alterations in immune function, by increasing central immune organ size and inducing a pro-inflammatory lymphocyte phenotype [32].

Therefore, inflammatory factors produced early in brain development either directly, by inducing MIA, or indirectly, by the maternal absence of a potent anti-inflammatory factor such as vitamin D may, represent a convergent pathway towards developmental brain abnormalities and psychiatric conditions later in life. Initial evidence for this hypothesis stems from previous observations showing prenatal poly(I:C)-induced MIA, and developmental vitamin D deficiency in rodents produce an overlapping spectrum of long-term behavioral abnormalities and early molecular changes in the fetal brains [33–37].

The objectives for this study were threefold. (a) We sought to verify whether autism-related behavioral deficits induced by poly(I:C) are present at a more translationally relevant prepubertal age. (b) Given the anti-inflammatory properties of vitamin D, the present study was designed to investigate whether use of calcitriol, [1α, 25-dihydroxy vitamin D3 (1,25OHD)], the hormonally active form of vitamin D, could prevent poly(I:C)-induced behavioral dysfunctions relevant to ASD, including deficits in social interaction, stereotyped behavior, innate anxiety, and emotional learning. (c) Does 1,25OHD block the poly(I:C)-induced inflammatory response in either dams or fetal brains?

## Methods

### Animals

C57BL6/N mice were used throughout the study. Female and male breeders were obtained from Charles River Laboratories (Sulzfeld, Germany) at the age of 12–14 weeks. Breeding began after 2 weeks of acclimatization to the animal holding rooms, which were temperature- and humidity-controlled (21 ± 1 °C, 55 ± 5%) facilities under a reversed light-dark cycle. All animals had ad libitum access to water and standard rodent chow (Kliba 3430, Kaiseraugst, Switzerland) which contains 1000-IU cholecalciferol/kg.

### Experimental groups

For the purpose of the maternal manipulations, C57BL6/N female mice were subjected to a timed-mating procedure as described previously [18]. Pregnant dams on gestation day (GD) 9 were first injected subcutaneously with 1,25OHD (Vit_D_) or vehicle (VEH) and were then immediately injected intravenously with either poly(I:C) (POL) or saline solution (CON) as described below. GD 9 in the mouse roughly corresponds to human gestational weeks 4 to 5 in terms of limbic neurogenesis (http://translatingtime.net/translate). This gestational window was selected based on epidemiological studies suggesting that the first trimester of human pregnancy may be associated with maximal vulnerability for viral infection-mediated neurodevelopmental disorders such as ASD [5] and schizophrenia [38]. Thus, the pregnant dams were divided into four treatment groups: CON/VEH, CON/VIT_D_, POL/VEH, POL/VIT_D_. For behavioral studies, pups remained with the same dam until weaning at 21 days of age, after which littermates of the same sex were caged separately and maintained in groups of 3–5 animals per cage. Each experimental group on postnatal day (PND) 35 consisted of offspring derived from multiple independent litters (at least six in each prenatal treatment condition) and included male subjects only to circumvent interpretative limitations arising from hormonal fluctuations in females [39]. For cytokine studies, a separate cohort of 18 GD9 pregnant dams received the appropriate solution and were decapitated 4 h after injection in order to collect dam blood and fetal brains for cytokine measurements (see below).

### Administration of 1,25OHD or vehicle

1,25OHD (Vit_D_) (solid powder, Calbiochem, EMD Millipore, Cat No 679101-50UG) was dissolved in absolute ethanol to 100 ng/μl as a stock solution. The stock was aliquoted and was stored in sterile tubes at −80 °C until further use. Subsequently, drug delivery solution was prepared as follows: 4 μl Vit_D_ (100 ng/μl) or absolute ethanol were added into 2 ml of corn oil to make a drug solution for delivery. The solution was vortexed to disperse Vit_D_ in the corn oil. The dosing was calculated with the formula 400 ng/kg/2 ml, such that the final injection volume in microliter was equal to 2 ml/kg dam body weight. The Vit_D_ or vehicle solution was administered subcutaneously in the neck region.

In pilot studies, we ensured the chosen dose would not induce any adverse effects on pup development or behavior as adults. To this end, 8 dams received vehicle and 9 dams Vit_D_ (400 ng/kg). These dams produced 50 and 55 offspring. In developing pups, we assessed body weight (g), crown-rump length (cm), eye opening, ear opening, ear folding, fur development, tooth eruption, and righting reflex on PND 8, 11, 14, 17, and 21 as outlined previously [40]. Vit_D_ had no effect on any of these measures (see Additional file 1). We also assessed dam weight gain, water and food consumption, and fecundity. Again there was no effect of Vit_D_ (see Additional file 2). We also assessed two behaviors in these offspring as adults. For both spontaneous locomotion in an open field (Additional file 3) and anxiety-like behavior in an elevated plus maze (Additional file 4) there was no effect of Vit_D_.

### Maternal immune activation during pregnancy

Poly(I:C) (potassium salt; Sigma-Aldrich, Buchs, St. Gallen, Switzerland; 5 mg/kg; calculated based on the pure form) or vehicle (saline) was dissolved in sterile pyrogen-free 0.9% NaCl (vehicle) solution to yield a final concentration of 1 mg/ml and was administered intravenously into the tail vein under mild physical constraint. The dose of poly(I:C) was selected based on previous dose–response studies [18].

### Behavioral studies

Behavioral testing was conducted in prepubertal offspring, between PND 30-40. For behavior, two different cohorts of animals were used. The use of two different cohorts served to minimize potential confounding factors associated with prolonged behavioral testing, but more importantly, to ensure that testing age remained in the prepubertal period. The first cohort was used for assessing innate anxiety behavior and social approach behavior, and the second cohort was used for assessing stereotypical behavioral and emotional learning and memory. For both cohorts, the number of subjects in each of the four experimental groups was *n* = 8. All behavioral testing was performed in the dark phase of the light-dark cycle.

### Elevated plus maze test

The elevated plus maze test served as a test for innate anxiety-like behavior. The apparatus was made of Plexiglas painted in gray and consisted of four equally spaced arms (5 × 30 cm^2^) radiating from a square center (5 × 5 cm^2^). One pair of opposing arms was enclosed with opaque walls (height: 15 cm) except for the side adjoining the central square. The remaining two arms were exposed with a parameter border (height: 3 mm) along the outer edges. The maze was elevated 70 cm above floor level and was positioned in a testing room with diffused lighting (approximately 20 Lux in open arm and 10 Lux in closed arm). A digital camera was mounted above the plus maze, captured images at a rate of 5 Hz and transmitted them to a PC running the EthoVision (Noldus Technology, Wageningen, The Netherlands) tracking system.

A test session began by placing the animal into the center zone with it facing one of the closed arms. It was then left to explore freely for 5 min before being returned to the home cage. After each trial, the apparatus was cleaned with water and dried before a new trial began. The (i) relative (percent) time spent in the open arms, (ii) distance moved in the open arms, and (iii) total distance moved in the open and closed arms during the entire 5-min test period were analyzed in order to index anxiety-related behavior. The percent time spent in the open arms was calculated using the formula [(time spent in the open arms)/(time spent in all arms) × 100].

### Social interaction

Social interaction was assessed using a social approach test in a modified Y-maze as previously outlined [21]. The apparatus was in the form of a Plexiglas Y-maze consisting of three identical arms (50 × 9 × 10 cm, length × width × height), radiating from a triangle center zone (each side 8 cm). Two of the three arms contained rectangular wire grid cages, the third arm served as the start zone.

Animals were first habituated to the apparatus by being allowed to explore the maze for 10 min on 2 days (day 1 and 2), which served to reduce novelty-induced locomotor hyperactivity. During the test day (day 3), one wire cage contained an unfamiliar C57BL/6N mouse (same sex as test mouse), and the other one contained a “dummy object” made of black LEGO™ (Billund, Denmark). The allocation of the objects (live mouse versus dummy object) was counterbalanced across arms and treatments. At the beginning of a test session, the animal was placed at the end of the start arm and was allowed to explore freely for 5 min. A camera, mounted above the maze, captured images and transmitted them to the EthoVision tracking system (Noldus, The Netherlands) to assess general locomotor activity. Social interaction was defined as nose orientation towards the wire cage within a 6-cm interaction zone adjacent to the wire cage. This was assessed by a trained experimenter who was blind to the treatment. After 5 min, the animal was removed and the apparatus was cleaned. Social interaction was assessed by analyzing the relative exploration time between the unfamiliar mouse and the inanimate dummy object using the following formula: % time spent with mouse = ([time spent with mouse]/[time spent with mouse] + [time spent with inanimate object]) × 100.

### Marble burying test

Stereotyped behavior was assessed using the marble burying test [41]. The test was performed as described in a study by Thomas and colleagues [42] with small modifications. Clean cages (27 × 16.5 × 12.5 cm) were filled with a 2-cm layer of chipped cedar wood bedding. Males were habituated to this cage for 10 min and then were returned to the home cage. Twenty colored glass marbles (15 mm diameter) were then gently laid on top of the bedding, equidistant from each other in a 4 × 5 arrangement. Animals were placed back into the testing cage, and the number of marbles buried (criteria for a buried marble was >50% marble covered by bedding material) in 10 min recorded.

### Cued Pavlovian fear conditioning

Emotional learning and memory were assessed using a cued Pavlovian fear conditioning test. The apparatus (Coulbourn Instruments, Allentown, PA, USA) has been fully described before [18]. It comprised two sets of test chambers to provide two distinct contexts (context A and B). The first set of chambers (context A) included operant chambers that were installed in ventilated, sound-insulated chests (72 cm wide × 45 cm long × 45 cm high). Each chamber of context A comprised a transparent rectangle measuring 14 × 16 × 30 cm (wide × long × high) and was fitted with a parallel grid shock floor (16 parallel bars, spaced 2 cm apart from each other; E10–18RF; Coulbourn Instruments), through which scrambled shocks could be delivered. Scrambled foot shocks provided the unconditioned stimulus (US). Illumination inside the chambers of context A was provided by a house light (2.8 W) positioned on the panel wall. The second set of chambers (context B) comprised cylinder transparent enclosures (16 cm diameter × 30 cm high), which rested on white Plexiglas floor instead of parallel grid floors. They were placed in ventilated, sound-insulated chests (72 × 45 × 45 cm) that were illuminated by an infrared light source instead of visible light. A fully automated algorithm was used to detect and quantify the freezing response as validated previously [43].

The test of cued Pavlovian fear conditioning included 3 phases, which were each separated by 24 h: The first day was conditioning (day 1, context A): following an initial habituation period of 6 min, the animals were exposed to three conditioning trials involving pairings between a conditioned stimulus (CS) and the US in context A. The CS was a 2.9-kHz tone measuring 86 dBA, which co-terminated with a 1-s, 0.3 mA foot shock US. The intertrial interval between each CS and US trial was 3 min. The amount of freezing during the three occasions of CS presentation provided a measure for the acquisition of fear conditioning. After conditioning, the animals were removed from the conditioning chambers and were brought back to their home cages 30 s after the last foot shock.

On the second day, the expression of conditioned fear expression towards context was examined (day 2, context A): the animals were returned to context A. They were placed in the test chamber for a period of 6 min without the presentation of the CS or US. This served as a test of conditioned fear expression towards the context, in which CS-US pairings took place. The expression of context freezing was indexed as percent time freezing across the 6-min period.

On the third day, CS-cued conditioned fear expression was examined (day 3, context B): the expression of conditioned fear towards the CS was assessed in a novel context B. Following an initial acclimatization period of 6 min, the CS was delivered (without subsequent shock presentation US) and remained on for 6 min, during which the time of conditioned freezing to the tone stimulus was evaluated. Conditioned freezing was expressed as percent time freezing.

### Inflammatory cytokine measurements

Systemic poly(I:C) treatment in pregnant mice is known to increase inflammatory cytokine levels in maternal and fetal compartments [7, 15]. Here, we tested the hypothesis that treatment with 1,25OHD may possibly reduce the production of such factors after maternal poly(I:C) treatment.

Pregnant mice on GD9 were assigned CON/VEH (*n* = 6), CON/VIT_D_ (*n* = 4), POL/VEH (*n* = 6), or POL/VIT_D_ (*n* = 6) treatment as described above. They were killed by decapitation 4 h post-treatment to collect maternal blood and fetal brains. The post-treatment interval was chosen based on previous findings showing peak cytokine responses at this interval [8, 44]. Trunk blood was collected into EDTA-containing tubes after decapitation. Tubes were gently inverted in order to mix blood with the EDTA. Plasma was collected upon centrifugation and aliquots were stored at −80 °C. Two fetal brains were collected from each dam as described before [8, 44]. They were weighed and immediately placed in 200 μl of ice cold lysis buffer (composed of 1 cocktail tablet—Complete Mini Protease Inhibitor Cocktail Tablets, Roche # 11836153001—dissolved in 10 ml lysis buffer—Roche: 04719956001), sonicated for 15 s, and then centrifuged at 12,000 rpm for 20 min at 4 °C [8, 44]. Supernatants were frozen at −80 °C until cytokine assays were performed.

Cytokine proteins in plasma and fetal brain homogenates were quantified using a customized Meso Scale Discovery (MSD) V-Plex electrochemiluminescence assay for mice, which allows ultralow detection of multiple cytokines in mouse plasma and supernatants [45]. In a sandwich ELISA V-plex plus, 96-well plates were coated with primary antibodies directed against interleukin IL-1β, IL-6, and TNF-α. Samples were added and cytokines were detected with corresponding detecting antibodies, which were prelabeled with SULFO-TAG™ (MSD, Rockville, MD, USA). The plates were read using the MESO SECTOR S 600 (MSD) imager and were analyzed using MSD’s Discovery Workbench analyzer and software package. All assays were run according to the manufacturer’s instructions.

### Vitamin D metabolite levels

Two vitamin D metabolites 24,25OHD3 and 25OHD3 were examined in the same maternal blood in which cytokines were analyzed using a liquid chromatography/tandem mass spectrometry method [46]. Therefore, this represents samples taken 4 h post 1,25OHD administration. The assay used was insensitive to the very low levels of 1,25OHD itself found in blood.

### Statistical analysis

All data were analyzed using parametric analysis of variance (ANOVA). A 2 × 2 (MIA × Vit_D_ treatment) ANOVA was used to analyze the dependent measures in the elevated plus maze, social interaction, and marble burying tests. In the fear conditioning test, acquisition of the conditioned fear response during the conditioning phase (day 1) was analyzed using 2 × 2 × 3 (MIA × Vit_D_ treatment × trial) repeated-measures ANOVA, whereas conditioned freezing towards the context (day 2) and CS-cued conditioned fear expression (day 3) were analyzed using 2 × 2 (MIA × Vit_D_ treatment) ANOVAs. Cytokines in the plasma of dams and in the fetal brain tissue and vitamin D metabolites in maternal blood were analyzed using a 2 × 2 (MIA × Vit_D_ treatment) ANOVA as well. Following these initial ANOVAs, Tukey’s multiple comparison test were conducted whenever appropriate. Statistical significance was set at *P* < 0.05. All statistical analyses were performed using the statistical software StatView (version 5.0) implemented on a PC running the Windows XP operating system.

## Results

### Innate anxiety-like behavior

**Fig. 1:**
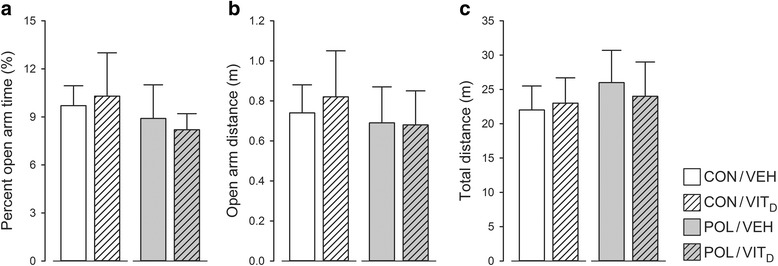
Elevated plus maze behaviors in juvenile offspring who were exposed to prenatal immune challenge (POL) or prenatal vitamin D treatment (Vit_D_). Neither MIA or Vit_D_ treatment alone nor their combination affected (**a**) time spent and (**b**) distance moved in the open arms of the elevated plus maze. Similarly, there were no group differences in (**c**) total distance moved. All values are mean ± SEM (*n* = 8)

Neither MIA or Vit_D_ treatment alone nor their combination affected (a) time spent and (b) distance moved in the open arms of the elevated plus maze. The relative time spent and distance moved in the open arms (Fig. 1a, b) were similar between the four experimental groups. Likewise, there were no group differences in terms of the total distance moved (Fig. 1c), suggesting that the prenatal manipulations did not affect general locomotor activity.

### Social interaction

**Fig. 2:**
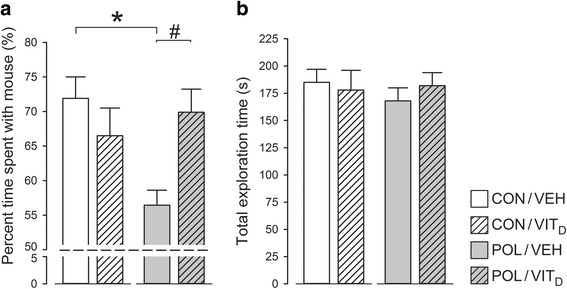
Social approach behavior in juvenile offspring who were exposed to prenatal immune challenge (POL) or prenatal vitamin D treatment (Vit_D_). **a** Compared with control mice poly(I:C)-treated offspring spent less time with a novel conspecific compared to an inanimate object (approaching chance level 50%) demonstrating impaired social approach behavior (*P < 0.05). This impairment was not evident when Vit_D_ was co-administered (#*P* = 0.06) suggesting Vit_D_ blocked the negative effects of MIA on social functions. **b** Prenatal exposures had no effects on locomotion in this test. All values are mean ± SEM (*n* = 8)

In the social interaction test, the relative time spent with an unfamiliar mouse was taken as a measure of social approach behavior. ANOVA of this measure revealed a significant interaction between MIA × Vit_D_ treatments (*F* [2, 28] = 4.305, *P* < 0.05). Subsequent post hoc comparisons verified that the percent time spent with the mouse was significantly (*P* < 0.05) decreased in POL/VEH relative to CON/VEH mice, demonstrating impaired social approach behavior following MIA (Fig. 2a). Intriguingly, the MIA-induced impairment in social approach behavior was not evident when Vit_D_ was co-administered (Fig. 2a), suggesting that 1,25OHD blocked the negative effects of MIA on social functions. These effects were not accompanied by changes in general locomotor activity as indexed by the total distance moved (Fig. 2b).

### Stereotyped behavior

**Fig. 3:**
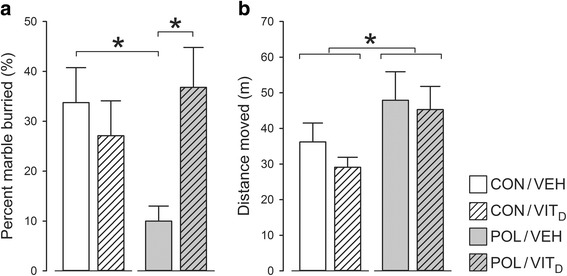
Marble burying behavior in juvenile offspring who were exposed to prenatal immune challenge (POL) or prenatal vitamin D treatment (Vit_D_). **a** Marble burying was markedly reduced in POL/VEH mice relative to all other groups, suggesting that MIA alters stereotyped behavior in prepubertal offspring (*P < 0.05). Vit_D_ co-administration completely blocked this MIA-induced alteration in marble burying (*P < 0.05). **b** An examination of total distance showed MIA treated mice moved significantly more in this test regardless of Vit_D_ co-treatment (*P < 0.05). All values are mean ± SEM (*n* = 8)

The percentage of marbles buried in the marble burying test is used as an index of stereotyped behavior. As summarized in Fig. 3a, this measure was markedly reduced in POL/VEH mice relative to all other groups, suggesting that MIA alters stereotyped behavior in prepubertal offspring. Importantly, Vit_D_ co-administration fully prevented the MIA-induced alterations in marble burying. These interpretations were supported by the presence of a significant interaction between MIA and Vit_D_ (*F* [1, 26] = 4.720, *P* < 0.05), and by the subsequent post hoc comparisons confirming a significant difference between POL/VEH and all other groups (all *P* < 0.05). An examination of total distance moved revealed a main effect of MIA (*F* [1, 28] = 4.808; *P* < 0.05), indicating that poly(I:C)-exposed mice moved significantly more in this test regardless of Vit_D_ co-treatment (Fig. 3b).

### Emotional learning and memory

**Fig. 4:**
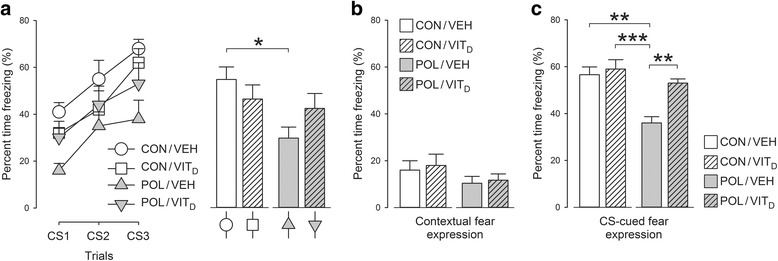
Acquisition of fear memory, association with context and fear memory behavior in juvenile offspring who were exposed to prenatal immune challenge (POL) or prenatal vitamin D treatment (Vit_D_). **a** MIA exposed mice had an impaired ability to acquire a conditioned fear response. The percentage time freezing to successive CS-US trials was significantly lower in poly(I:C)-treated mice compared to controls (*P < 0.05). This MIA-induced reduction in fear acquisition was not evident when Vit_D_ was co-administered suggesting that vitamin D blocked the effects of MIA on emotional learning. **b** Prenatal treatment had no effect on context-cued conditioned fear expression. **c** Poly(I:C)-exposed mice had reduced expression of CS-cued conditioned fear when re-exposed to the CS-tone; relative to CON/VEH (***P < 0.001) and relative to CON/VIT_D_ or POL/VIT_D_ (**P < 0.01). Hence, maternal exposure to Vit_D_ appeared to normalize the deficit in CS-cued conditioned fear in MIA animals. All values are mean ± SEM (*n* = 8)

The acquisition of the fear response during the conditioning phase (day 1) was used as an index of emotional learning towards aversive stimuli. The development of conditioned fear was influenced by both MIA and Vit_D_ treatment, as supported by the significant main effect of MIA (*F* [1, 28] = 6.922, *P* < 0.05) and its interaction with Vit_D_ treatment attaining statistical trend level (*F* [1, 28] = 4.092, *P* = 0.052). Indeed, from percentage time freezing to successive CS-US trials was significantly lower in POL/VEH mice as compared to CON/VEH mice (*P* < 0.05, Fig. 4a). The MIA-induced reduction in fear acquisition was not evident when Vit_D_ was co-administered (Fig. 4a), suggesting that vitamin D blocked the effects of MIA on emotional learning.

1 day after conditioning, the animals were placed back to the same conditioning chambers to test the expression of conditioned fear towards the context, in which CS-US pairings took place. There were no group differences in this measure (Fig. 4b), suggesting that the prenatal manipulation did not affect context-cued conditioned fear expression. Interestingly, however, the expression of CS-cued conditioned fear during the tone test, which took place 1 day after the context test, was significantly decreased in POL/VEH mice compared to all three other groups (Fig. 4c). ANOVA revealed a significant interaction between MIA and Vit_D_ treatment (*F* [1, 28] = 4.546, *P* < 0.05), and subsequent post hoc comparisons confirmed a significant difference between POL/VEH and CON/VEH or CON/VIT_D_ (P’s < 0.001) and between POL/VEH and POL/VIT_D_: *P* < 0.01). Hence, maternal exposure to Vit_D_ appeared to normalize the deficit in CS-cued conditioned fear in MIA animals.

### Cytokine measurements in maternal plasma and fetal brains

**Fig. 5:**
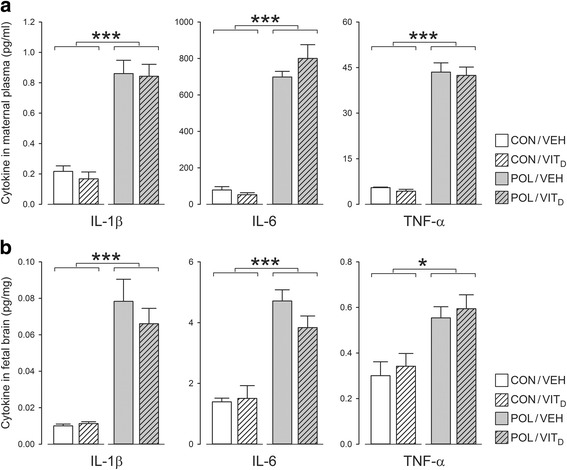
Cytokine levels in maternal blood and fetal brain 4 h post poly(I:C) exposure. **a** Poly(I:C) led to a marked increase in the maternal levels of all pro-inflammatory cytokines measured (all ***P < 0.001). Vit_D_ co-administration had no effect on MIA-induced maternal pro-inflammatory cytokine production. **b** Poly(I:C) also led to a significant increase in fetal brain pro-inflammatory cytokines, as supported by the main effect of MIA for IL-1β and IL-6 (***P < 0.001) and TNF-α (*P < 0.05). Again, Vit_D_ co-administration did not affect basal or MIA-induced elevations of pro-inflammatory cytokines. All values are mean ± SEM, (*n* = 6) for CON/VEH, POL/VEH, and POL/VIT_D_ groups and (*n* = 4) for the CON/VIT_D_ group

As expected [8, 44], administration of poly(I:C) led to a marked increase in the maternal levels of the pro-inflammatory cytokines with a main effect of MIA for IL-1β (*F* [1, 18] = 87.324, *P* < 0.001), IL-6 *F* [1, 18] = 203.262, *P* < 0.001) and TNF-α *F* [1, 18] = 274.450, *P* < 0.001) (Fig. 5a). Vit_D_ co-administration had no effect on MIA-induced maternal pro-inflammatory cytokine production (Fig. 5a).

MIA also led to a significant increase in fetal brain pro-inflammatory cytokines, as supported by the main effect of MIA for IL-1β (*F* [1, 18] = 20.13, *P* < 0.001), IL-6 (*F* [1, 18] = 46.225, *P* < 0.001) and TNF-α (*F* [1, 18] = 7.567, *P* < 0.05) (Fig. 5b). Again, Vit_D_ co-administration did not affect basal or MIA-induced elevations of pro-inflammatory cytokines in the fetal brain (Fig. 5b).

#### Vitamin D metabolite levels

There was no main effect of 1,25OHD or poly(I:C) treatment on maternal levels of either the major circulatory form of vitamin D 25OHD or its inactive metabolite 24,25OHD when measured 4 h after exposure; 25OHD and 24,25OHD concentrations (nM) in each group were as follows: CON/VEH 29.7 ± 7.4, 21.2 ± 5.5, CON/VIT_D_ 40.3 ± 11.0, 27.2 ± 6.7, POL/VEH 35.3 ± 11.4, 20.3 ± 8.2, and POL/VIT_D_ 36.8 ± 8.8, 23.6 ± 4.1; (*P* > 0.1).

## Discussion

Maternal infection and/or inflammation is a recognized environmental risk factor for developmental brain disorders, including ASD [2, 5, 6, 47]. MIA in experimental animals reproduces various behavioral phenotypes in offspring of potential relevance to autism [9, 19–21]. In view of the known immunomodulatory and neuroprotective effects of Vit_D_ [34] and the fact that Vit_D_ deficiencies have been linked to autism [26]. The present study tested the hypothesis that maternal administration of the active Vit_D_ hormone, 1,25OHD, would prevent autism-relevant behavioral abnormalities in prepubertal MIA offspring. Our data support this hypothesis by showing that maternal Vit_D_ co-administration blocked the emergence of the ASD-relevant deficits in social interaction, stereotyped behavior, and emotional learning and memory. The additional assessment of maternal and fetal inflammatory markers, however, suggested that these beneficial effects are unlikely to be attributable to anti-inflammatory mechanisms.

### Vit_D_ effects on MIA-induced ASD-related behaviors

Our study demonstrates that prenatal immune challenge with poly(I:C) on GD9 disrupts social approach behavior in prepubertal offspring. This finding corroborates the results of previous studies reporting impaired social approach behavior in adult offspring who were subjected to poly(I:C)-induced MIA [9, 19–21, 48]. Our findings showing a prepubertal onset of social interaction deficits are an important extension to this previous data in adults. First, it increases the face validity of poly(I:C)-based MIA models for ASD, which is characterized by deficiencies in social interaction starting early in childhood. Second, these findings highlight that MIA-induced impairments in social interaction are an early pathological manifestation preceding many other behavioral and cognitive dysfunctions in this model which do not appear until early adulthood [49]. Such adult-onset behaviors include deficits in sensorimotor gating [14, 50], selective attention [15, 16], and working memory [51]. Finally, in the context of this study, an even more important finding was that maternal treatment with the active vitamin D hormone 1,25OHD was capable of preventing this altered social behavior in juvenile MIA offspring.

Another main finding of our study was that MIA induced a decrease in the acquisition of conditioned fear in juvenile offspring. This phenotype likely represents an amygdala-driven deficit in associative emotional learning [52, 53] and has been similarly observed in other models relevant to ASD. For example, prenatal exposure to valproic acid has been linked to an increased incidence of ASD and is widely used to model ASD-related pathologies in animals [54]. Offspring from rat dams prenatally exposed to certain doses of valproate have very similar deficits in the acquisition of conditioned fear [55]. When such profound deficits in fear acquisition are present, it is also likely to affect the expression of learned fear [52, 53]. This was the case in our study, where juvenile MIA offspring were found to display a marked deficit in CS-cued conditioned fear expression. MIA did not, however, affect the expression of conditioned fear towards the context, suggesting that hippocampal processing of contextual cues in fear memory was largely intact in juvenile MIA offspring [52, 53]. Importantly, the MIA-induced deficits in the acquisition *and* expression of CS-cued fear conditioning were prevented by co-administration of Vit_D_, adding further evidence for the hypothesis that prenatal Vit_D_ treatment in the event of MIA may be beneficial against the development of ASD-related abnormalities.

Interestingly, previous studies using poly(I:C)-based MIA models have found *increased* expression of CS-cued conditioned fear when the offspring reached adulthood [21]. These abnormalities are thus diametrically opposite to those manifest in juvenile MIA offspring, which show *impaired* acquisition and expression of CS-cued fear conditioning. Even though we do not know the underlying mechanisms for these divergent manifestations, they may tentatively be related to altered maturation of relevant neuronal substrates such as the amygdala. Indeed, longitudinal studies in MIA models emphasize the importance of altered brain maturation in precipitating age-dependent behavioral and cognitive dysfunctions [50, 51, 56].

In addition to analyzing social behavior and fear-related learning and memory, the present study also assessed digging behavior. To this end, a marble burying task was performed. Juvenile poly(I:C) offspring showed a significant reduction in stereotyped digging behavior compared to saline-treated control offspring. This reduced digging was clearly not due to any MIA-induced impairment in motor function in the poly(I:C) offspring, because these animals were shown to be mildly hyperactive in this task. Again importantly this abnormal stereotyped behavior was prevented by maternal Vit_D_ administration. Other authors have described poly(I:C)-induced deficits in adult offspring with this task [19, 20]. However, when tested in adulthood, MIA offspring show an *increase* in marble burying [19, 20], which is typically interpreted as an increase in stereotyped behavior [57]. Despite this, *reduced* marble burying has been reported in no less than four distinct mouse models with deletions in prominent genetic pathways closely associated with ASD [58]. Most experiments to date confirm that marbles do not represent an aversive stimulus for animals [59]. Rather, marble burying is a measure of normal stereotyped behavior in rodents; given sufficient bedding depth, mice display stereotyped digging behavior in this environment and tend to cover or bury objects as part of this ethologically normal digging behavior [41]. Whilst an increase is typically interpreted as an ASD-related increase in stereotyped digging [57], a decrease can just as easily represent an impairment in normal stereotyped behavior. The younger age examined here may also represent an important experimental variable that has been little explored in this task. In any case, the marble burying task provided further evidence for the preventive potential of Vit_D_ against the development of MIA-induced behavioral abnormalities.

Some of these behavioral abnormalities, i.e., cognitive deficits reported in MIA juveniles may also have translational relevance to other psychiatric disorders such as schizophrenia. However, the fact that MIA with poly(I:C) has induced abnormalities in social interaction and cognition at a more translationally relevant age, i.e., juveniles, together with the presence of behavioral abnormalities not normally associated with schizophrenia such as altered digging behavior suggest to us that this animal model may have greater relevance to ASD

### Vit_D_ does not alter maternal or fetal inflammatory cytokine production

In view of the known immunomodulatory effects of vitamin D, an obvious neuroprotective mechanism explored here was the possibility that Vit_D_ ameliorates the inflammatory response induced by poly(I:C). Anti-inflammatory approaches have been successful for other MIA-focused interventions. For instance, it has been demonstrated that co-administration of an antibody directed against one of the major inflammatory factors associated with poly(I:C)-induced MIA, IL-6, prevents most of the behavioral deficits caused by poly(I:C). Similarly, in mice where the IL-6 receptor is genetically ablated many poly(I:C) induced behaviors are also blocked [7]. More recently, a similar approach with antibodies directed at another crucial cytokine associated with poly(I:C)-induced MIA, IL-17a, has again shown this approach to be successful in ameliorating MIA-induced behavioral deficits [9]. Thus, in an attempt to understand how Vit_D_ might modulate poly(I:C)-induced behavioral abnormalities, we investigated whether Vit_D_ might reduce the expression of key inflammatory factors. As expected, we found large increases in the pro-inflammatory cytokines IL-1β, IL-6, and TNFα in maternal blood and fetal brains 4 h after MIA. This is in line with previous studies demonstrating that maternal immune challenge with poly(I:C) enhances these pro-inflammatory cytokines in maternal sera or plasma and fetal brain tissues at similar post-treatment intervals [8, 14, 44]. However, co-administration of Vit_D_ had no effect on these cytokines in any of the treatment groups, strongly suggesting that the ASD-preventive potential of this hormone at least in this MIA model is not primarily related to its anti-inflammatory effects.

The immunomodulatory actions of Vit_D_ on factors such as inflammatory cytokines are mediated via the toll-like receptors (TLR) 2/4 [60]. For instance, numerous studies have shown that Vit_D_ exerts its anti-microbial actions via TLR2/4 receptors [60] and modulates cytokine secretion from human dendritic cells towards an anti-inflammatory environment [61]. Inflammatory agents upregulate both the Vit_D_ receptor and the CYP271b which is the primary enzyme responsible for the production of the active vitamin D hormone via a TLR2/4 mechanism, indicating the close reciprocal feedback between Vit_D_ and these receptors [60, 61]. Given that RNA viruses and artificial double-stranded RNA constructs such as poly(I:C) act via primarily a different toll-like pathway, i.e., TLR3, perhaps explains at least in part why Vit_D_ failed to block MIA-induced inflammation.

### What are other potential 1,25OHD-mediated neuroprotective mechanisms in fetal brain?

Because our study does not find evidence for anti-inflammatory actions underlying the preventive potential of prenatal Vit_D_ administration, we consider it important to draw attention to some other potential Vit_D_-mediated mechanisms that might be operating after maternal immune challenge. Earlier we described how the behavioral phenotype of poly(I:C)-exposed offspring was ameliorated/abolished by focusing on therapies that directly target inflammatory cytokine production/signaling. However, poly(I:C) can also adversely impact the early brain via (a) impairing brain cell differentiation; (b) reducing the availability of trace metals crucial for normal brain development; (c) reducing the availability of nerve growth factor (NGF). Importantly, as detailed below, these same abnormalities are both reproduced by animal models of DVD-deficiency and reversed by Vit_D_.

In fetuses derived from mid-gestational poly(I:C)-exposed dams, there is an increased subcortical proliferation [8, 50]. There are also abnormalities in cortical laminae formation coincident with changes in cell division in the postnatal brain. In particular postnatal day 10, poly(I:C) offspring have an increase in late dividing cells in outer layers of the cortex [62]. The DVD-deficient brain is also hyperproliferative [33, 63, 64]. Vit_D_ has been shown to be a potent anti-proliferative agent in virtually every tissue examined to date. We have shown that the addition of Vit_D_ reduces hyperproliferation in cells from DVD-deficient brains ex vivo in the form of neurospheres [63]. Therefore, one plausible neuroprotective action of Vit_D_ may be to diminish poly(I:C)-induced hyperproliferation in the early fetal brain.

The robust increase in IL-1β and IL-6 induced by poly(I:C) also serves to increase peripheral synthesis of binding proteins for trace elements such as iron and zinc. This is an immunoprotective action reducing the availability of these elements which are essential for the invading pathogens [65, 66]. However, iron and zinc are also essential for normal brain development. Therefore, reducing iron and zinc availability may have adverse consequences. Importantly, in other models of MIA, comorbid anemia produces worse outcomes [32]. In addition, supplementation with iron prevents the appearance of several phenotypes in MIA offspring [11]. Maternal zinc supplementation also prevents the effects of MIA on later cognitive performance in adult offspring [12]. Vit_D_ is known to regulate the absorption of iron, zinc, and many other trace metals from the gut [67]. Indeed, anemia and Vit_D_ deficiency have been closely linked for decades [68]. Most recently, Vit_D_ has been shown to directly regulate a crucial gene in zinc transport SLC30A10. Transcription of this gene was under the direct control of SLC30A10 promoter associated ligand-bound VDR. Moreover, the transporting protein itself, ZnT10 was dramatically upregulated by Vit_D_ [69]. We therefore postulate that a second plausible neuroprotective action of Vit_D_ may be to restore the absorption of such trace elements normally reduced by poly(I:C).

The neurotrophin NGF is reduced in the placenta 24 h after MIA with poly(I:C) [70]. We have previously shown that NGF is reduced in the fetal DVD-deficient brain [33]. We and others have also demonstrated that Vit_D_ reliably upregulates NGF in neurons. Against these backgrounds, we postulate that a third plausible neuroprotective action of Vit_D_ may be to restore NGF to normal levels in the poly(I:C)-treated fetal placenta and brain.

Finally, given that MIA and maternal vitamin D deficiency are both risk-modifying factors for autism and given that the distinct overlap in behavioral phenotypes produced in the animal models of these environmental risk factors, we addressed the issue of whether MIA induces vitamin D deficiency. Obviously, if this were the case then the corrective actions of the active vitamin D hormone may simply be treating an MIA-induced vitamin D deficiency. However, our results clearly show no effect of poly(I:C) on both major circulatory measures of vitamin D, ruling out this possibility.

## Conclusions

MIA using poly(I:C) is becoming more widely used as an animal model that produces behavioral phenotypes of relevance to ASD. Our work here extends the face validity of this model as we show that ASD-relevant behavioral alterations emerge already in prepubertal animals. Importantly, our work strongly supports a protective role for the hormonal form of Vit_D_ when administered during gestation against MIA-induced changes in ASD-related behaviors. Vit_D_ deficiency itself is increasingly being linked with ASD [27]. Furthermore, there is initial evidence to suggest that Vit_D_ supplementation in children with ASD may be effective in treating this condition [71]. Unfortunately, 1,25OHD as the active Vit_D_ hormone cannot be used in pregnancy due to its potential hypercalcaemic effects on the developing fetus. However, our findings suggest that future studies with the safe-to-use dietary form of Vit_D_, cholecalciferol, are warranted. If dietary supplementation with cholecalciferol was shown to be successful in the prevention of MIA-induced behavioral abnormalities relevant to ASD (and related neurodevelopmental disorders), then this may open new avenues for the establishment of novel therapeutic public health preventative interventions in a similar manner to the use of folate to prevent spina bifida.

## Additional files

Additional file 1: A table describing the absence of any effect of Vit_D_ on various aspects of pup development. (DOCX 20 kb)
Additional file 2: A graph describing the absence of any effect of Vit_D_ on various aspects of dam physiology, fecundity, and pup survival. Assessment of (A) body weight gain (g), (B) water, and (C) food consumption for dams who were exposed to prenatal vehicle (VEH) (*n* = 8) or prenatal vitamin D treatment (Vit_D_) at GD9 (*n* = 9). (D) Litter size and (E) pup survival rate (%) in the 72 h post birth were monitored for the treated groups. In all measures, Vit_D_ exposure at GD9 leads to no significant effect on GD11, GD13, and GD15 comparing to vehicle injected dams. All values are mean ± SEM. (TIFF 624 kb)
Additional file 3: A graph describing spontaneous locomotion behavior of adult offspring in a novel open field. There is no effect of developmental exposure to Vit_D_. Spontaneous locomotion in an open field in adult offspring who were exposed to prenatal vehicle (VEH) (*n* = 50) or prenatal vitamin D treatment (Vit_D_) (*n* = 55). Neither VEH nor Vit_D_ treatment affected distance traveled in the open field. All values are mean ± SEM. (TIFF 4380 kb)
Additional file 4: A graph describing anxiety-like behavior in an elevated plus maze in adult offspring. There is no effect of developmental exposure to Vit_D_. Elevated plus maze behaviors in adult offspring who were exposed to prenatal vehicle (VEH) (*n* = 50) or prenatal vitamin D treatment (Vit_D_) (*n* = 55). Neither VEH nor Vit_D_ treatment affected the time spent in the closed arms, open arms, and center of the elevated plus maze. (B) Similarly, there were no group differences in distance moved in this test. All values are mean ± SEM. (TIFF 324 kb)

## Abbreviations

1,25OHD: 1α,25-dihydroxy vitamin D3
24,25OHD: 24,25-Dihydroxycholecalciferol
25OHD: 25-hydroxyvitamin D
ASD: Autism spectrum disorder
CS: Conditioned stimulus
DVD: Developmental vitamin D
LC/MS/MS: Liquid chromatography/mass spectrometry/mass spectrometry
MIA: Maternal immune activation
MSD: Meso Scale Discovery
NGF: Nerve growth factor
PBMC: Peripheral blood mononuclear cell
PND: Postnatal day
Poly(I:C): Polyriboinosinic-polyribocytidylic acid
TLR: Toll-like receptor
VDR: Vitamin D receptor
Vit_D_: 1α,25-dihydroxy vitamin D3
