# Supplementary material for: Vitamin D treatment during pregnancy prevents autism-related phenotypes in a mouse model of maternal immune activation
Source: Mol Autism. 2017 Mar 7;8:9. doi: 10.1186/s13229-017-0125-0 (PMC5351212; doi:10.1186/s13229-017-0125-0)
Supplement: Additional file 1: — A table describing the absence of any effect of VitD on various aspects of pup development. (DOCX 20 kb) [file 13229_2017_125_MOESM1_ESM.docx]

**Additional file 1: Table S1.** The physiology of the developing pups were assessed, including body weight (g), crown-rump length (cm), eye opening, ear opening, ear folding, fur development, tooth eruption and righting reflex on PND8, PND11, PND14, PND17 and PND21 as following previously method [39]. In all measures, Vit_D_ exposure to the dams at GD9 produced no adverse developmental outcome. All values are mean ± SD.

|  |  |  |  |  |  |  |  |  |  |  |
| --- | --- | --- | --- | --- | --- | --- | --- | --- | --- | --- |
|  | PND8 | | PND11 | | PND14 | | PND17 | | PND20 | |
|  | VEH | VIT_D_ | VEH | VIT_D_ | VEH | VIT_D_ | VEH | VIT_D_ | VEH | VIT_D_ |
| Body weight (g) | 4.03+0.31 | 4.09+0.46 | 5.20+0.33 | 5.25+0.57 | 6.46+0.43 | 6.52+0.65 | 7.04+0.57 | 7.27+0.74 | 8.49+0.79 | 8.87+0.94 |
| Rump length (cm) | 4.27+0.20 | 4.32+0.28 | 4.94+0.19 | 4.98+0.23 | 5.90+0.21 | 5.94+0.35 | 6.35+0.51 | 6.34+0.49 | 7.04+0.43 | 7.04+0.47 |
| Eye opening | 0.00+0.00 | 0.00+0.00 | 0.34+0.40 | 0.17+0.31 | 2.63+0.55 | 2.74+0.61 | 4.68+0.43 | 4.81+0.37 | 5.00+0.00 | 4.99+0.07 |
| Ear opening | 0.00+0.00 | 0.00+0.00 | 0.55+0.85 | 0.59+0.84 | 3.08+0.42 | 3.06+0.59 | 4.34+0.58 | 4.34+0.54 | 4.82+0.24 | 4.94+0.16 |
| Ear folding | 0.00+0.00 | 0.06+0.23 | 1.57+0.79 | 1.67+0.87 | 3.45+0.55 | 3.18+0.52 | 4.51+0.46 | 4.37+0.46 | 4.81+0.27 | 4.89+0.21 |
| Fur development | 1.10+0.45 | 1.02+0.52 | 2.83+0.30 | 2.79+0.43 | 4.03+0.52 | 3.87+0.58 | 4.40+0.43 | 4.30+0.45 | 4.75+0.25 | 4.74+0.25 |
| Maxillary eruption | 0.00+0.00 | 0.00+0.00 | 0.00+0.00 | 0.06+0.23 | 2.03+0.61 | 2.18+0.74 | 3.26+0.60 | 3.19+0.68 | 4.39+0.47 | 4.44+0.55 |
| Mandibular eruption | 0.22+0.38 | 0.15+0.35 | 1.47+0.66 | 1.46+0.81 | 3.15+0.58 | 3.22+0.42 | 3.86+0.33 | 3.85+0.43 | 4.65+0.30 | 4.70+0.28 |
| Righting reflex | 1.45+0.75 | 1.35+0.72 | 2.90+0.54 | 2.71+0.55 | 3.99+0.40 | 3.88+0.49 | 4.99+0.08 | 5.00+0.00 | 4.98+0.11 | 5.00+0.00 |
