# Supplementary figures and images for: Vitamin D treatment during pregnancy prevents autism-related phenotypes in a mouse model of maternal immune activation
Source: Mol Autism. 2017 Mar 7;8:9. doi: 10.1186/s13229-017-0125-0 (PMC5351212; doi:10.1186/s13229-017-0125-0)

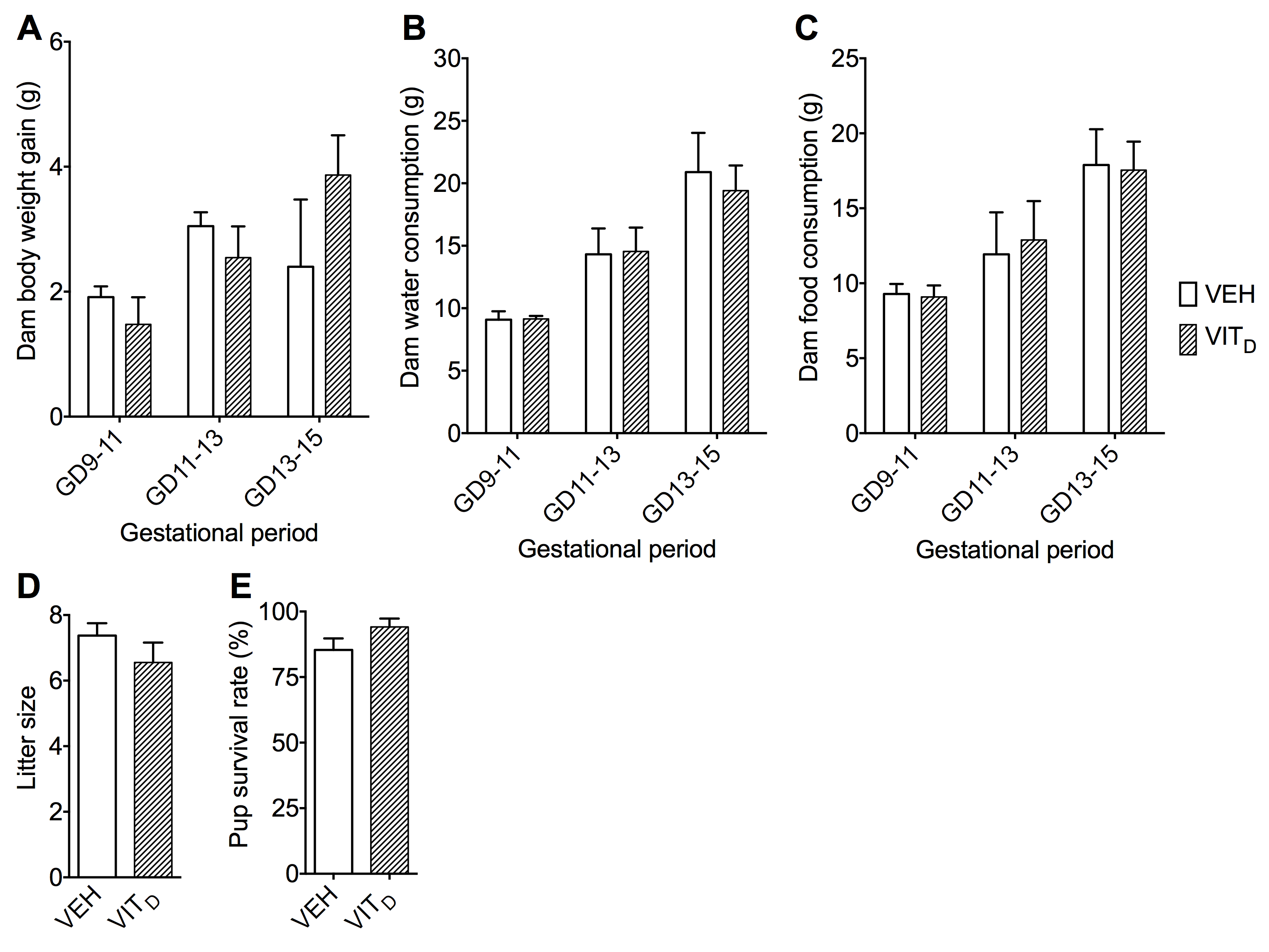

Supplement: Additional file 2: — A graph describing the absence of any effect of VitD on various aspects of dam physiology, fecundity, and pup survival. Assessment of (A) body weight gain (g), (B) water, and (C) food consumption for dams who were exposed to prenatal vehicle (VEH) (n = 8) or prenatal vitamin D treatment (VitD) at GD9 (n = 9). (D) Litter size and (E) pup survival rate (%) in the 72 h post birth were monitored for the treated groups. In all measures, VitD exposure at GD9 leads to no significant effect on GD11, GD13, and GD15 comparing to vehicle injected dams. All values are mean ± SEM. (TIFF 624 kb) [file 13229_2017_125_MOESM2_ESM.tiff]

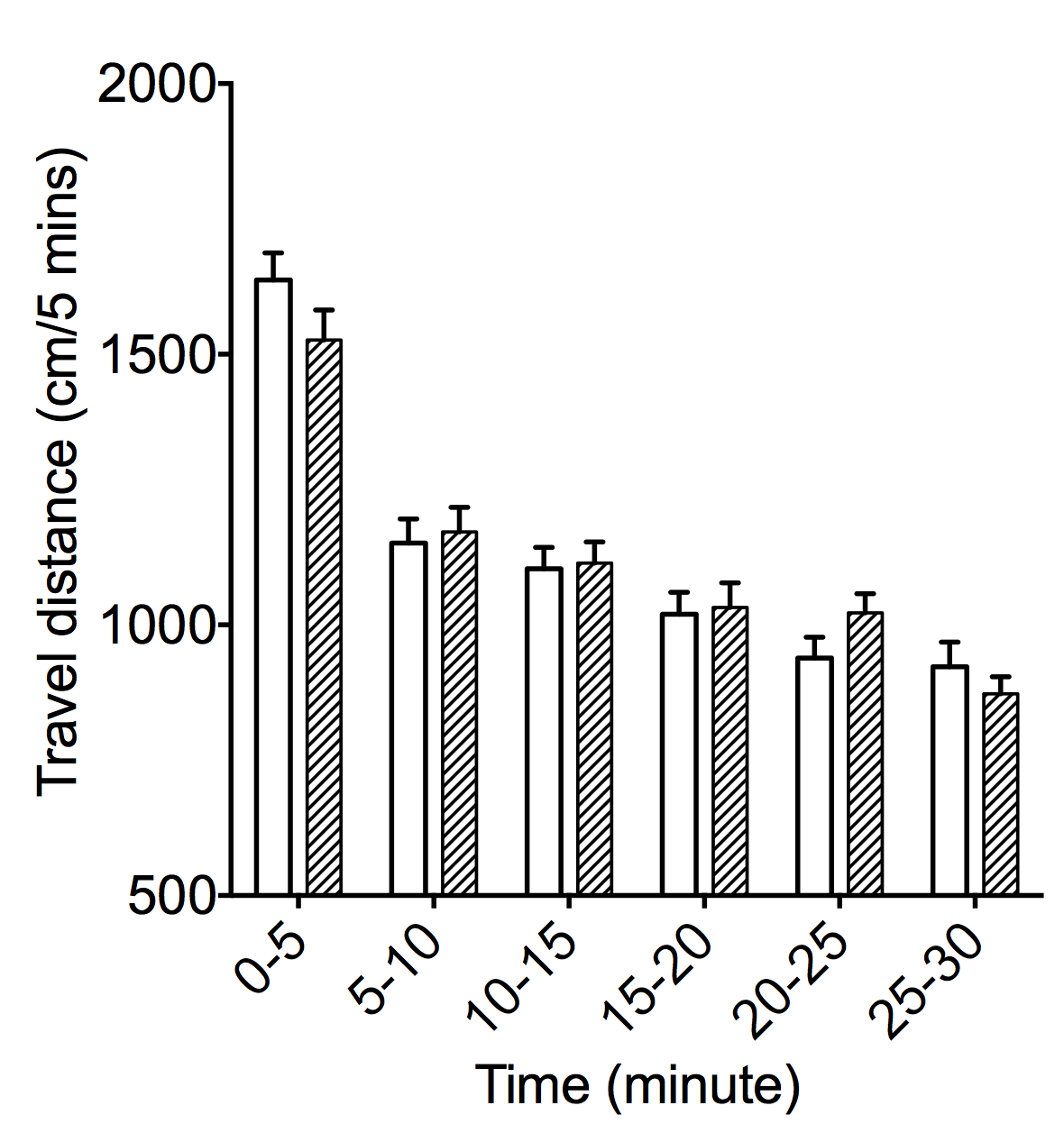

Supplement: Additional file 3: — A graph describing spontaneous locomotion behavior of adult offspring in a novel open field. There is no effect of developmental exposure to VitD. Spontaneous locomotion in an open field in adult offspring who were exposed to prenatal vehicle (VEH) (n = 50) or prenatal vitamin D treatment (VitD) (n = 55). Neither VEH nor VitD treatment affected distance traveled in the open field. All values are mean ± SEM. (TIFF 4380 kb) [file 13229_2017_125_MOESM3_ESM.tiff]

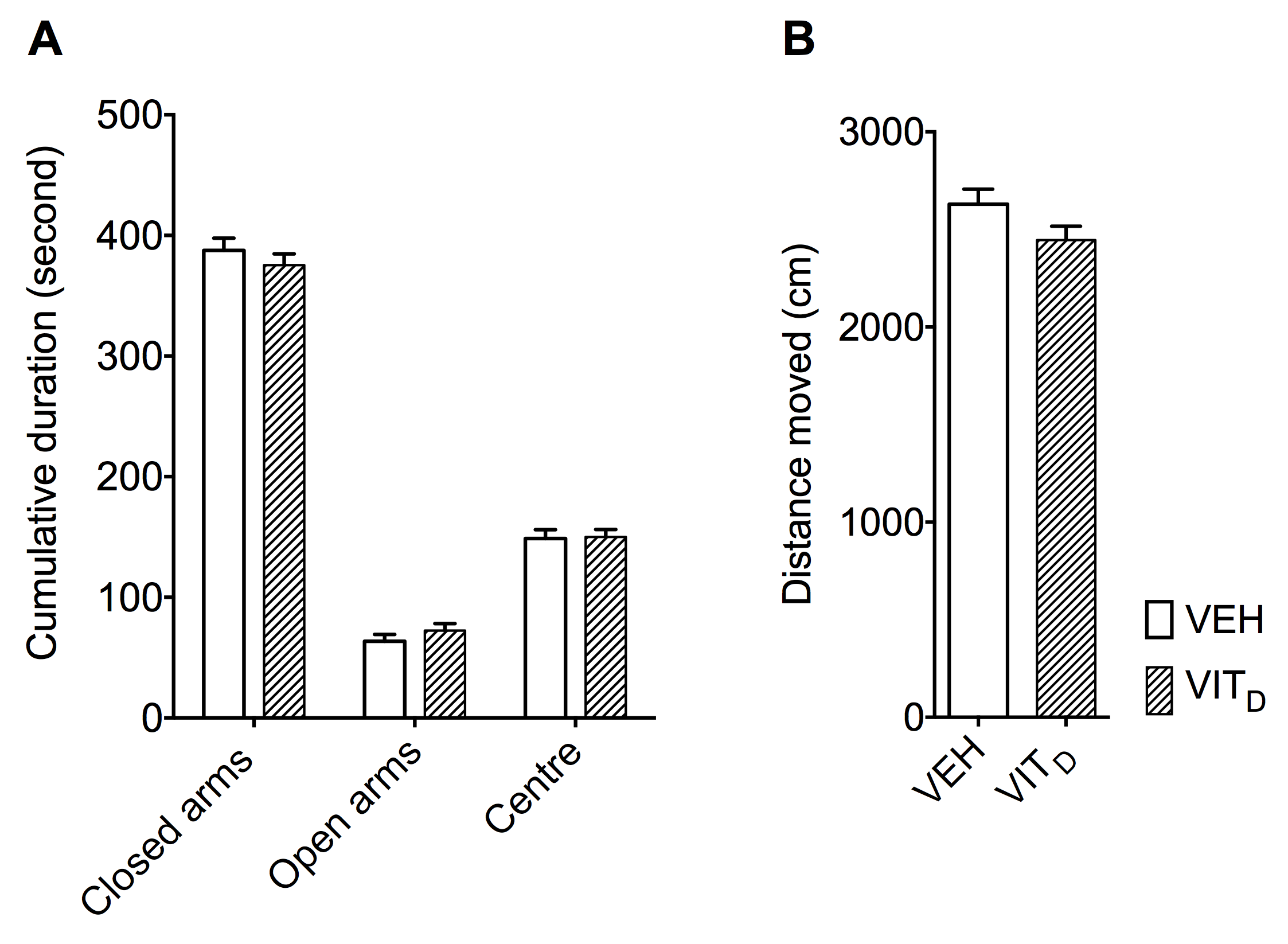

Supplement: Additional file 4: — A graph describing anxiety-like behavior in an elevated plus maze in adult offspring. There is no effect of developmental exposure to VitD. Elevated plus maze behaviors in adult offspring who were exposed to prenatal vehicle (VEH) (n = 50) or prenatal vitamin D treatment (VitD) (n = 55). Neither VEH nor VitD treatment affected the time spent in the closed arms, open arms, and center of the elevated plus maze. (B) Similarly, there were no group differences in distance moved in this test. All values are mean ± SEM. (TIFF 324 kb) [file 13229_2017_125_MOESM4_ESM.tiff]
